# Tofacitinib in early active axial spondyloarthritis: protocol of a randomized double-blind, placebo-controlled, multicenter phase IV study, FASTLANE

**DOI:** 10.1177/1759720X251324429

**Published:** 2025-03-12

**Authors:** Valeria Rios Rodriguez, Lidia Sánchez-Riera, Hildrun Haibel, Caroline Höppner, Murat Torgutalp, Fabian Proft, Judith Rademacher, Elke Binder, Annette Diehl, Ivana Vranic, Yuxi Zhao, Rajiv Mundayat, Arne Yndestad, Denis Poddubnyy

**Affiliations:** Department of Gastroenterology, Infectious Diseases and Rheumatology (including Nutrition Medicine), Charité—Universitätsmedizin Berlin, Hindenburgdamm 30, Berlin 12203, Germany; Pfizer SLU, Madrid, Spain; Department of Gastroenterology, Infectiology and Rheumatology (including Nutrition Medicine), Charité—Universitätsmedizin Berlin, Berlin, Germany; Department of Gastroenterology, Infectiology and Rheumatology (including Nutrition Medicine), Charité—Universitätsmedizin Berlin, Berlin, Germany; Department of Gastroenterology, Infectiology and Rheumatology (including Nutrition Medicine), Charité—Universitätsmedizin Berlin, Berlin, Germany; Department of Gastroenterology, Infectiology and Rheumatology (including Nutrition Medicine), Charité—Universitätsmedizin Berlin, Berlin, Germany; Department of Gastroenterology, Infectiology and Rheumatology (including Nutrition Medicine), Charité—Universitätsmedizin Berlin, Berlin, Germany; Pfizer SLU, Madrid, Spain; Pfizer Inc., Cambridge, MA, USA; Pfizer Inc., Tadworth, UK; Pfizer Inc., Cambridge, MA, USA; Pfizer Inc., New York, NY, USA; Pfizer Inc., Oslo, Norway; Department of Gastroenterology, Infectiology and Rheumatology (including Nutrition Medicine), Charité—Universitätsmedizin Berlin, Berlin, Germany; Toronto University Hospital, Toronto, ON, Canada

**Keywords:** axial spondyloarthritis, clinical trial, magnetic resonance imaging, tofacitinib, treatment

## Abstract

**Background:**

Early treatment initiation is one of the strongest predictors of good treatment response in axial spondyloarthritis (axSpA). Recently, the Assessment in SpondyloArthritis International Society (ASAS) defined early axSpA as a diagnosis of axSpA with a duration of axial symptoms equal to or less than 2 years. Tofacitinib is a Janus kinase (JAK) inhibitor for the treatment of ankylosing spondylitis.

**Objectives:**

Compare the efficacy and safety of tofacitinib versus placebo (both on non-steroidal anti-inflammatory drug (NSAID) background) in patients with active early axSpA and inadequate response to at least one NSAID.

**Design:**

This is a phase IV, randomized, double-blind, placebo-controlled, multicenter clinical trial.

**Methods and analysis:**

The study will recruit 104 patients aged ⩾18 and ⩽45 years with active early axSpA (chronic back pain ⩽2 years), inadequate response to at least one NSAID, and objective signs of active inflammation (on magnetic resonance imaging (MRI) of sacroiliac joints (SIJs) or elevated C-reactive protein). Patients will be randomized 1:1 to receive tofacitinib 5 mg twice daily or placebo, with background naproxen 500 mg twice daily for 16 weeks. Patients not meeting early treatment response criteria at week 4 will receive open-label tofacitinib until week 16. Primary and key secondary endpoints at week 16 will be the proportion of patients achieving disease remission (Axial Spondyloarthritis Disease Activity Score <1.3) and change from baseline in MRI SIJ Spondyloarthritis Research Consortium of Canada osteitis score, respectively. Safety will be monitored up to 4 weeks after the last study drug dose.

**Ethics:**

The study will be performed according to the ethical principles of the Declaration of Helsinki and will be approved by independent ethics committees of each center.

**Discussion:**

This is one of the first randomized clinical trials designed to evaluate the efficacy and safety of a JAK inhibitor in the recently ASAS-defined “early” axSpA population.

***Trial registration*:** ClinicalTrials.gov: NCT06112665; CTIS: 2023-505050-18-00.

## Introduction

Axial spondyloarthritis (axSpA) is a chronic inflammatory rheumatic disease, primarily affecting the spine and sacroiliac joints (SIJ), and is characterized by symptoms such as chronic back pain and stiffness, with eventually arthritis and enthesitis. In addition, patients can present extra-musculoskeletal manifestations such as uveitis, psoriasis, or inflammatory bowel disease. Patients can be classified into two categories: radiographic axSpA (r-axSpA) also named ankylosing spondylitis (AS), with visible structural damage on the SIJ on radiographic imaging; and non-radiographic axSpA (nr-axSpA), without such radiographic structural changes in the SIJ. The global prevalence of axSpA varies among different geographic regions, ranging from 0.3% to 1.4%.^1^

In recent years, the concept of “early axSpA” has gained attention, referring to patients in the initial phase of the disease. Definitions of “early” have been based on different aspects, with some researchers focusing on the duration of the disease and others on the absence of irreversible structural damage.^2^ The Assessment of SpondyloArthritis International Society (ASAS) aimed to establish a consensus and standardized definition for the term “early axSpA” based on evidence and expert opinion^2^ to bring consistency to the study populations in research focused on patients with axSpA at the early stage of the disease. This definition for early axSpA was approved in January 2023 during the Annual ASAS Meeting as follows: “patients with a diagnosis of axSpA with a duration of axial symptoms equal to or less than 2 years. Axial symptoms should include spinal/buttock pain or morning stiffness; regardless of the presence/absence of radiographic damage and should be considered by a rheumatologist as related to axSpA.”^3^

The treatment of patients with axSpA has evolved along with the advancements in diagnosis, and the recommendations for their management have been recently updated by the ASAS/European Alliance of Associations for Rheumatology.^4^ Non-steroidal anti-inflammatory drugs (NSAIDs) are still considered the first-line therapy, followed by treatment with tumor necrosis factor (TNF) inhibitor, interleukin-17 (IL-17) inhibitor, or Janus kinase (JAK) inhibitor if high disease activity persists despite previous treatment with NSAIDs or when intolerance or contraindications to NSAIDs exist (current practice is to start with a TNF inhibitor or IL-17 inhibitor).^4^

Tofacitinib is an oral JAK inhibitor and a targeted synthetic disease-modifying antirheumatic drug (tsDMARD), that has been investigated for the treatment of several immune-mediated diseases^5–10^ and is approved for adult patients with rheumatoid arthritis, psoriatic arthritis, ulcerative colitis, AS, and patients 2 years and over with polyarticular juvenile idiopathic arthritis and juvenile psoriatic arthritis.^11,12^ In the pivotal phase III study in patients with active AS (including those who were bDMARD naïve or had a previous history of TNF inhibitor use), tofacitinib demonstrated superior clinical efficacy, with rapid and sustained response across disease activity measures and quality of life-related outcomes.^9^ Further research supported these findings, showing that tofacitinib reduces spinal and SIJ inflammation, as measured by the Spondyloarthritis Research Consortium of Canada (SPARCC) MRI score after 12 weeks of treatment in patients with AS, and is associated with greater clinical responses.^13^ Recently, data from phase II/III and phase III multicenter, randomized, double-blind, placebo-controlled clinical trials evaluating Upadacitinib, another drug from the JAK inhibitor family, have demonstrated benefit in patients with active axSpA (radiographic and non-radiographic), supporting further use of this mode of action in this disease area.^14,15^

Growing evidence supports the benefits of early treatment in axSpA, positioning early intervention not only as one of the strongest predictors for good treatment response in axSpA^16^ but also as a potential predictor of drug-free remission.^17,18^ Studies such as the ESTHER study (axSpA patients with less than 5 years of symptom duration) and the INFAST study (axSpA patients with less than 3 years of disease duration) suggested a higher probability of remission.^17,18^ In these studies, the remission rate reached approximately 50%, notably higher than the 7%–16% observed in studies with patients with advanced disease stages, as evidenced in pivotal phase III studies with TNF inhibitors,^19–23^ IL-17 inhibitors,^24–26^ and JAK inhibitors.^9,14^ Thus, the proposed study aims to address the following important and so far, unclear aspects of the treatment of axSpA: (i) what is the probability of remission achievement with an advanced therapy in patients with early axSpA (up to 2 years of symptom duration) and (ii) is there a difference in efficacy between an NSAID given alone (as the standard of conventional therapy) and a combination of tsDMARD and NSAID at this early disease stage?

## Methods

### Aim and objectives

The main objective of this study is to evaluate the efficacy of tofacitinib versus placebo (both given on a background of NSAID treatment) in achieving disease remission at week 16 in patients with active early axSpA (back pain ⩽2 years) who have had an inadequate response to at least one NSAID. In addition, the efficacy of tofacitinib versus placebo in reducing MRI inflammation in the SIJ, early treatment response from weeks 1 to 4 (including the effect on patient-reported outcomes (PROs)), and very early (within the first week) response on pain reduction, as well as safety and tolerability of tofacitinib compared to placebo will be evaluated.

### Study population and design

This is a randomized, double-blind, placebo-controlled multicenter study.

The study population will consist of patients aged ⩾18 and ⩽45 years who have a clinical diagnosis of axial SpA and fulfill the ASAS classification criteria for axSpA (classified as either non-radiographic or radiographic according to ASAS criteria) with back pain duration ⩽2 years and inadequate response to at least one NSAID. In addition, for inclusion in the study, patients will be required to show high disease activity defined as Bath Ankylosing Spondylitis Disease Activity Index (BASDAI) ⩾4 and back pain ⩾4 (corresponding to BASDAI Q2, on a numerical rating scale (NRS)) plus either elevated (>5 mg/L) C-reactive protein (CRP) or active inflammation on MRI of the SIJ. Patients are required to be naïve to any biologic or targeted synthetic DMARDs. The list of key inclusion and exclusion criteria is shown in Table 1.

**Table 1.** Key inclusion and exclusion criteria for the FASTLANE study.

Inclusion criteria
• Subject ⩾18 and ⩽45 years of age at the screening visit. • Written informed consent. • Clinical diagnosis of axSpA. • Fulfilment of ASAS classification criteria for axSpA. • Symptom (back pain) duration for ⩽2 years, according to the definition of “early axial SpA” by ASAS. • Active disease activity as defined by: (a) BASDAI ⩾4 and back pain score (BASDAI Question 2) of ⩾4 (on a 0–10 NRS) at screening and baseline. and (b) Objective signs of inflammation at screening, evident by: (i) MRI with SIJ inflammation (assessed by two central readers) and/or (ii) Elevated serum CRP levels (>5 mg/L). • History of an inadequate response to at least one NSAID, other than naproxen.
Exclusion criteria
• Current or past treatment with biologic or targeted synthetic DMARD. • Contraindications for MRI or treatment with tofacitinib. • Any active significant acute or chronic infection (at the discretion of the investigator). • History of disseminated/multidermatomal herpes zoster/simplex or history of recurrent herpes zoster. • Patients positive for HIV, hepatitis B, or C at randomization. • Pregnant, breastfeeding, or plan to become pregnant during the study women. • Current malignancy or history of malignancies except adequately treated or excised basal cell or squamous cell carcinoma or cervical carcinoma in situ. • Live vaccinations within 6 weeks prior to baseline.

ASAS, Assessment of SpondyloArthritis International Society; axSpA, axial spondyloarthritis; BASDAI, Bath Ankylosing Spondylitis Disease Activity Index; CRP, C-reactive protein; DMARD, disease-modifying antirheumatic drug; HIV, human immunodeficiency virus; MRI, magnetic resonance imaging; NRS, numerical rating scale; NSAID, non-steroidal anti-inflammatory drug; SIJ, sacroiliac joint.

**Figure 1.**
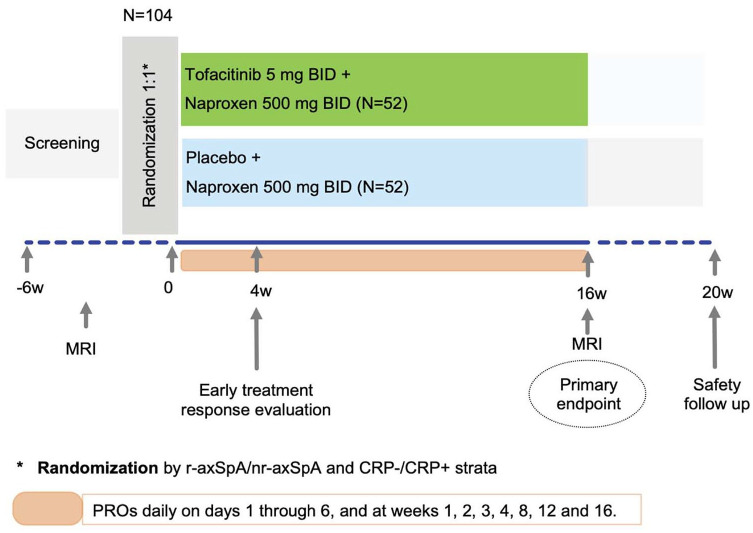
Study Design of FASTLANE study.

The study will include a 6-week screening period, a 16-week treatment period, and a safety follow-up at week 20. During the screening period, patients will be assessed for study eligibility and will undergo procedures outlined in the assessment schedule (Table 2). The baseline MRI of the SIJ will be performed within this period to assess the presence of active inflammation (bone marrow edema/osteitis) compatible with axial SpA (as assessed by two independent central readers, and an additional independent reader in case of disagreement) according to ASAS definition for positive MRI (“active sacroiliitis”).^27^ At baseline, eligible patients will be randomized on a 1:1 ratio by r-axSpA/nr-axSpA and elevated CRP/non-elevated CRP strata to receive either tofacitinib 5 mg two times daily (BID) plus naproxen 500 mg BID or matching tofacitinib placebo plus naproxen 500 mg BID for a 16-week double-blind treatment period (Figure 1). After the baseline visit, face-to-face clinical assessment will take place at weeks 4 and 16. An evaluation for early treatment response will be performed at week 4. Patients who maintain high disease activity according to BASDAI ⩾4 and demonstrate <20% improvement in BASDAI from baseline at week 4 will receive tofacitinib 5 mg BID open-label until week 16. After the last tofacitinib dose at week 16 or in case of early termination (only in patients who remained on treatment for at least 4 weeks during the study), primary, key secondary, and other efficacy secondary endpoints will be assessed. MRI of the SIJ will be performed in a maximum time window of ±7 days from the last visit.

**Table 2.** Study procedures and timelines of the FASTLANE study.

Study procedures	Screening ⩽6 weeks	Baseline week 0	Week 4^a^	Week 16^a^/ET^b^	Week 20^a^/FU
	Visit 1	Visit 2	Visit 3	Visit 4	Visit 5 (phone)
Informed consent	X				
Inclusion/exclusion criteria	X	X^c^			
Randomization		X			
Demographics	X				
Medical/surgical history including VTE and cardiovascular risk assessment (SCORE2)	X	X^c^	X	X	
Contraception check^d^	X	X	X	X	X
Vaccination status	X				
Prior/concomitant medication	X	X	X	X	X
Vital signs^e^	X	X	X	X	
Weight and height, BMI		X			
Smoking assessment^f^	X	X	X	X	
EMMs ASAS report	X	X	X	X	
Physical examination	X	X	X	X	
44 TJC/SJC		X	X	X	
Enthesitis assessment (MASES)		X	X	X	
Dactylitis count		X	X	X	
PGA (on a 0–10 NRS)	X	X	X	X	
ASDAS_CRP_		X	X	X	
BASDAI (on a 0–10 NRS)^g^	X	X	X	X	
BASFI (on a 0–10 NRS)		X	X	X	
ASAS HI		X	X	X	
FACIT-F		X	X	X	
PtGA (on a 0–10 NRS)^g^	X	X	X	X	
Nocturnal back pain in the last week (on a 0–10 NRS)^g^		X	X	X	
Total pain, total back pain, and nocturnal back pain in the last 24 h (on a 0–10 NRS)^g^		X	X	X	
BASMI, chest expansion		X	X	X	
Study drug administration		X	X	X	
Hematology, blood chemistry^h^	X	X	X	X	
Lipid profile (fasting samples)^i^	X	X	X	X	
CRP	X	X	X	X	
HLA-B27 test^j^	X				
Urine pregnancy test^k^	X	X	X	X	
HIV, Hepatitis B, and Hepatitis C serologies	X				
QuantiFERON^®^-TB or T-SPOT^®^ TB (or similar IGRA test)^l^	X				
Chest X-ray^l^	X				
Pelvis X-ray^m^	X				
MRI of SIJs	X			X	
ECG	X				
Adverse events		X	X	X	X

a Weeks after the first intake of the study drug, visits have a time window of ±7 days.

b ET = early termination visit for subjects who prematurely discontinue the study for any reason.

c Interim history to check for investigator’s assessment of benefit:risk.

d For pre-menopausal female subjects and non-sterile males only.

e Body temperature, heart rate, blood pressure.

f Former and actual smoking state at screening, pack-years calculation for the former and actual smoker.

g Home-based collection of PROs at Weeks 1, 2, 3, 8, and 12. Total pain, total back pain, and nocturnal back pain in the last 24 h will also be collected daily on days 1–6. PROs at Week 0 will be collected during Visit 2, at Week 4 during Visit 3, and at Week 16 during Visit 4.

h Complete blood count, total bilirubin, ALT, AST, GGT, AP, creatinine, and CK.

i Total cholesterol, LDL cholesterol, HDL cholesterol, and triglycerides.

j To be performed only if there is no HLA-B27 test previously performed (previous results need to be demonstrated by a laboratory or medical report).

k Female patients of child-bearing potential only. High sensitivity urine pregnancy test. A serum pregnancy test is to be performed in case of doubts. A pregnancy test will be performed in all site study visits.

l QuantiFERON^®^-TB, T-Spot^®^ TB test, or similar IGRA test and Chest X-ray performed within 3 months prior to screening will be accepted. In female patients of child-bearing potential, evidence of a negative pregnancy test prior to chest X-ray is required.

m Pelvis X-rays; to be performed at screening only if there is no image performed within 6 months prior to screening and/or previous available imaging does not meet the pre-specified quality requirements. In female patients of child-bearing potential, evidence of a negative pregnancy test prior to pelvis X-ray is required.

ALT, alanine aminotransferase; AP, alkaline phosphatase; ASAS, Assessment of Spondyloarthritis International Society; ASAS HI, ASAS health index; ASDAS, Axial Spondyloarthritis Disease Activity Score using C-reactive protein; AST, aspartate aminotransferase; BASDAI, Bath Ankylosing Spondylitis Disease Activity Index; BASFI, Bath Ankylosing Spondylitis Functional Index; BASMI, Bath Ankylosing Spondylitis Metrology Index; BMI, body mass index; CK, creatinine kinase; CRP, C-reactive protein; ECG, electrocardiogram; EMM, extra-musculoskeletal manifestation; ET, early termination; FACIT-F, Functional Assessment of Chronic Illness Therapy-Fatigue; FU, follow up; GGT, gamma-glutamyl transferase; HIV, human immunodeficiency virus; HLA-B27, human leukocyte antigen B27; MASES, Maastricht Ankylosing Spondylitis Entheses Score; MRI, magnetic resonance imaging; NRS, numerical rating scale; PGA, Physician Global Assessment; PROs, patient-reported outcomes; PtGA, Patient Global Assessment; SCORE2, Systemic Coronary Risk Estimation 2; SJC, swollen joint count; TB, tuberculosis; TJC, tender joint count; VTE, venous thromboembolism.

Digitally collected PROs will include total pain, total back pain, and nocturnal back pain in the last 24 h on days 1–6. From week 1, digitally collected PROs will be collected weekly from weeks 1 to 3, and then at weeks 8 and 12; and will also include BASDAI, nocturnal back pain, and patient global assessment (PtGA) during the last 7 days. PROs at screening (BASDAI, PtGA only), baseline, week 4, and week 16 will be collected during the correspondent face-to-face visit.

Safety will be monitored throughout the whole duration of the study up to 4 weeks after the last study drug dose. A comprehensive cardiovascular (CV) and venous thromboembolism (VTE) risk assessment will be performed at each applicable face-to-face visit to maintain an adequate benefit:risk balance for all study participants. For VTE, subjects will be screened for factors such as heart failure, recent myocardial infarction, inherited coagulation disorders, prior VTE, hormone therapy use, malignancies, major surgery, or immobilization. Additional risk factors including age, obesity, diabetes, hypertension, smoking, and family history of VTE will also be considered. Any newly developed risks will prompt reevaluation and possible treatment discontinuation. Similarly, the CV risk assessment will include evaluation using the Systemic Coronary Risk Estimation 2 calculator, along with other factors like atherosclerotic CV disease, uncontrolled diabetes, smoking history, and family history of premature CV events. Continuous monitoring at each visit will ensure timely management of newly emerging risks, promoting patient safety.

This protocol has been developed using the SPIRIT (Standard Protocol Items: Recommendations for Interventional Trials) 2013 statement, a guideline on standard protocol items for clinical trials.^28^ SPIRT Checklist for FASTLANE study is available as Supplementary Material.

### Endpoints

The primary endpoint is the proportion of patients achieving an Axial Spondyloarthritis Disease Activity Score^29^ with CRP (ASDAS) <1.3 at week 16.

The key secondary endpoint is the change from baseline in the MRI SIJ SPARCC osteitis score at week 16. Other efficacy secondary endpoints are the proportion of patients achieving (1) ASDAS low disease activity (<2.1) at weeks 4 and 16; (2) ASDAS clinically important improvement (⩾1.1) at weeks 4 and 16; (3) ASDAS major improvement (⩾2.0) at weeks 4 and 16; (4) ASAS20 and ASAS40 response and ASAS partial remission at weeks 4 and 16; (5) BASDAI50 at weeks 4 and 16; (6) change from baseline in ASDAS, Physician Global Assessment, and serum CRP at weeks 4 and 16; and (7) change from baseline in BASDAI and PtGA at weeks 1, 2, 3, 4, 8, 12, and 16. In addition, the efficacy of tofacitinib 5 mg BID versus placebo on achieving early treatment response will be assessed by the proportion of subjects requiring treatment escalation to open-label tofacitinib at week 4. Additional secondary endpoints will include treatment responses on pain reduction (including very early response assessed daily within the first week of treatment), function, mobility, peripheral musculoskeletal manifestations, fatigue, quality of life, and extra-musculoskeletal manifestations at week 16 and at all other applicable time points. Safety secondary endpoints include incidence of adverse events (AEs), AE of special interest, and AEs leading to study discontinuation. Exploratory endpoints are the development of structural changes in the MRI of SIJ by the change from baseline in the SPARCC structural score of the MRI SIJs at week 16, and to assess the efficacy of tofacitinib 5 mg BID versus placebo by subgroup populations. The full list of study endpoints is found in Table 3.

**Table 3.** Primary, secondary, and exploratory endpoints of the FASTLANE study.

*Primary endpoint*
Proportion of subjects achieving ASDAS <1.3 at week 16.
*Key secondary endpoint*
Change from baseline in the MRI SIJ SPARCC osteitis score at week 16.
*Other secondary endpoints*
At weeks 4 and 16, the proportion of patients achieved: • an ASDAS clinically important improvement • an ASDAS major improvement • ASDAS <2.1 • ASAS20, ASAS40, ASAS partial remission, BASDAI50 responses At weeks 4 and 16 vs baseline, improvement in disease activity, function, axial mobility, and QoL was measured according to: • ASDAS • BASDAI • PGA • CRP • BASFI • BASMI and chest expansion • 44 TJC/SJC for peripheral involvement • MASES for enthesitis • Dactylitis count • ASAS-HI and FACIT-F The proportion of subjects requiring treatment escalation to open-label tofacitinib at week 4 (proportion of subjects with BASDAI ⩾4 plus BASDAI reduction <20% from baseline at week 4) At weeks 1, 2, 3, 4, 8, 12, and 16 vs baseline, improvement in: • total back pain (NRS) (question 2 of BASDAI) in the last 24 h and 7 days • nocturnal back pain (NRS) in the last 24 h and 7 days • total pain (NRS) in the last 24 h • PtGA At day 1–6 vs baseline, improvement in: • Total back pain (NSR), nocturnal back pain (NSR), and total pain (NSR) in the last 24 h At week 16 vs baseline, improvement in EMMs (overall and by EMM type) At week 20 (or 4 weeks after ET), safety and tolerability of tofacitinib vs placebo by incidence of AEs, TEAEs, AEs of special interest,^a^ AEs leading to study discontinuation.
*Tertiary/exploratory endpoints*
At week 16, the proportion of patients achieving ASDAS <1.3 by: • radiographic status (nr-axSpA/r-axSpA) subgroup • CRP (elevated/non-elevated) subgroup • sex subgroup (female/male) • tofacitinib early treatment response subgroup (responders at week 4/non-responders at week 4) At week 16 vs baseline, improvement in MRI SIJ SPARCC structural score.^b^ At week 16 vs baseline, improvement in MRI SIJ SPARCC osteitis score by: • radiographic status (nr-axSpA/r-axSpA) subgroup • CRP (elevated/non-elevated) subgroup • sex subgroup (female/male) • tofacitinib early treatment response subgroup (responders at week 4/non-responders at week 4).

a AEs of special interest are: all malignancies excluding NMSC, NMSC, MACE, DVT, PE, ATE, GI perforation, hepatic events, DILI, HZ, OI, OI excluding HZ, SIE, and ILD.

b Components include backfill, fat metaplasia, sclerosis, erosion, and ankylosis.

AE, adverse events; ASAS, Assessment in SpondyloArthritis international Society; ASAS HI, ASAS Health Index; ASDAS, Axial Spondyloarthritis Disease Activity Score; ATE, arterial thromboembolism; BASDAI, Bath Ankylosing Spondylitis Disease Activity Index; BASFI, Bath Ankylosing Spondylitis Functional Index; BASMI, Bath Ankylosing Spondylitis Metrology Index; CRP, C-reactive protein; DILI, drug-induce liver injury; DVT, deep vein thrombosis; EMM, extra-musculoskeletal manifestations; FACIT-F, Functional Assessment of Chronic Illness Therapy- Fatigue; GI, gastrointestinal; HZ, herpes zoster; ILD, interstitial lung disease; MACE, major adverse cardiovascular events; MASES, Maastricht Axial Spondyloarthritis Enthesitis Score; MRI, magnetic resonance imaging; NMSC, non-melanoma skin cancer; nr-axSpA, non-radiographic axial spondyloarthritis; NRS, numerical rating scale; OI, opportunistic infections; PE, pulmonary embolism; PGA, Physician Global Assessment; PtGA, Patient Global Assessment; r-axSpA, radiographic axial spondyloarthritis; SIE, serious infections events; SIJ, sacroiliac joints; SJC, swollen joint count; SPARCC, Spondyloarthritis Research Consortium of Canada; TEAE, treatment-emergent adverse event; TJC, tender joint count.

### Site monitoring and data management

The collection of the data will be done electronically using electronic case report forms (eCRF). At every stage of the data management process, the data collected will be securely stored and patient confidentiality will be always maintained in accordance with data protection laws. Access to the EDC system will be by username/password combination only and available to authorized personnel. When data entry is performed at the investigator site, investigators will not have access to the whole database but only to the data entered by the individual site on their enrolled patients. The patient identification list will remain in the investigator’s file. A trained site monitor will conduct a site initiation visit, regular follow-up with the sites during the study, and a site closeout visit at the end. The site monitor will ensure that local teams and investigators are well-trained in the protocol and address any issues identified. To strengthen the reliability of the data, automatic validation checks will be performed for any irregularities in the eCRF entries and will allow modifications by the investigators until the lock of the database. In addition, the site monitor will conduct source data verification with selected data to detect errors and query inconsistencies.

This study may be audited by the sponsor, the marketing authorization holder of tofacitinib (i.e., Pfizer), any person authorized by the sponsor, or the competent health authority to determine the authenticity of the recorded data and compliance with the study protocol. In addition, changes to the protocol can only be made in a written protocol amendment format, which needs the approval of the sponsor and regulatory authorities before its implementation.

### Sample size

The sample size calculation is based on the primary efficacy endpoint (i.e., the proportion of patients achieving remission defined as ASDAS <1.3 at week 16). The sample size estimation is driven by treatment effect evaluation between an active treatment group and a placebo group. We assumed that tofacitinib (+naproxen) would be at least as effective as infliximab (plus naproxen) in this study population. Based on the results of the INFAST study,^30^ we anticipate that 20% in the placebo (+naproxen) group and 50% in the tofacitinib (+naproxen) group will achieve remission (ASDAS <1.3) at week 16. With these assumptions, a total of 104 subjects will be randomly assigned to one of the arms (tofacitinib 5 mg BID or placebo) in a ratio of 1:1 stratified by radiographic status (r-axSpA or nr-axSpA) and CRP status (elevated or non-elevated) to show the difference between the study arms with a power of 90% and two-sided alpha of 0.05. Considering low historical drop-out rates, the sample size will not be adjusted for the drop-out rate.

### Methods against bias

To mitigate bias, patients will undergo randomization during their baseline visit. They will be assigned to receive either tofacitinib 5 mg BID or placebo (both on a background of naproxen 500 mg BID), with assignments following a 1:1 ratio, stratified by radiographic classification (r-axSpA or nr-axSpA) and CRP status (elevated or non-elevated). The study will be conducted in a double-blind fashion to ensure an unbiased assessment of the clinical outcomes. In addition, MRI and X-ray of the SIJ will be assessed centrally by three trained readers (two independent readers, and an additional independent reader in case of disagreement) blinded to all clinical data including treatment allocation as well as to the time points for MRI SIJ image acquisition. Investigators and patients will remain blinded to the original randomization group allocation until the database release.

### Statistical analysis

The unblinded analysis will be performed on all patient data after the last patient has completed week 20 (or the last visit in early termination) and after the study database is locked.

Efficacy analyses will be performed in the modified intent-to-treat population that will be defined as all patients who are randomly assigned to study intervention with the investigational medicinal product (IMP, i.e., tofacitinib or placebo) and who have taken at least one dose of IMP. Participants will be analyzed using the treatment groups as they are randomized.

The primary analysis will focus on the proportion of patients who achieve remission, as defined by ASDAS <1.3 at the end of week 16. For comparison of tofacitinib versus placebo, the Mehrotra and Railkar^31^ method will be used, adjusting for the stratification factors (nr-axSpA/r-axSpA and elevated CRP/non-elevated CRP) if sufficient subjects are present in each stratum. Otherwise, the exact Chan and Zhang^32^ method will be applied. The difference in the remission rates between treatment groups and the corresponding two-sided 95% confidence interval adjusted for stratification factors will be reported. Missing values from patients who drop out or receive prohibited medication or treatment escalation for any reason before week 16 will be imputed as non-responders. For sensitivity analysis, the above analysis will be repeated using data as observed without missing imputation.

For the key secondary endpoint, change from baseline in MRI SIJ SPARCC osteitis score at week 16 will be analyzed using analysis of covariance adjusting for baseline score and stratification factor (if there are a sufficient number of subjects in each stratum, otherwise stratification factor will be removed from the model) to estimate the effect of initially randomized treatment. The least-squares means and the difference in least-squares means between treatment groups, both with their corresponding 95% confidence intervals will be presented. Data after patients receiving prohibited medicine or treatment escalation will be excluded from the analysis and only observed data will be used (missing data will not be imputed).

For continuous secondary endpoints measured at a single visit, the same analysis as defined in the key secondary endpoint analysis will be repeated. For endpoints with multiple visits, change from baseline over time will be analyzed using the mixed-effect model for repeated measures accounting for fixed effects of baseline score, visit, treatment, treatment-by-visit interaction, and stratification factors (if sufficient patients in each stratum). For sensitivity analysis of key secondary endpoints and secondary continuous endpoints, missing data will be imputed using baseline observation carried forward and the above corresponding analysis will be repeated.

For binary secondary endpoints, the same analysis as defined in primary analysis and corresponding sensitivity analysis will be applied accordingly. Safety analyses will include all patients randomly assigned to study intervention and who take at least one dose of IMP. Participants will be analyzed according to the treatment they have actually received. AEs will be coded using the Medical Dictionary for Regulatory Activities. Safety data will be presented in tabular and/or graphical format and summarized descriptively, where appropriate.

## Discussion

This trial addresses a relevant gap in the management of early axSpA, particularly in those patients with axial symptoms up to 2 years, as recently defined by the ASAS. Despite the growing recognition that early treatment is a key factor in preventing disease progression, there is currently very little evidence regarding the efficacy of advanced therapies, such as JAK inhibitors, in the early phases of axSpA.

The value of this study relies on its focus on early intervention and the new target population. Prior studies, like ESTHER and INFAST, have reported higher remission rates in patients with shorter disease duration in patients treated with TNFi. Regarding JAK inhibitors, tofacitinib has demonstrated efficacy in the treatment of AS; however, the potential for rapid and maintained disease control in early axSpA has not been researched in detail yet.

In addition, the inclusion criteria, which require signs of active inflammation on SIJ MRI and/or elevated CRP, ensure the selection of patients with objective evidence of disease activity, bringing robustness to the findings of the study. These results have the potential to impact clinical practice, supporting earlier and more targeted therapeutic strategies to prevent disease progression and improve long-term outcomes in early axSpA.

## Conclusion

This study is among the first to evaluate the efficacy and safety of the JAK inhibitor tofacitinib in patients with early active axSpA, a newly defined target population with a symptom duration of up to 2 years. While tofacitinib has demonstrated efficacy in AS, the value of this study lies in its focus on early intervention and the potential for rapid, sustained disease control in early axSpA, an area that remains underexplored.

## Supplemental Material

sj-doc-1-tab-10.1177_1759720X251324429 – Supplemental material for Tofacitinib in early active axial spondyloarthritis: protocol of a randomized double-blind, placebo-controlled, multicenter phase IV study, FASTLANE Supplemental material, sj-doc-1-tab-10.1177_1759720X251324429 for Tofacitinib in early active axial spondyloarthritis: protocol of a randomized double-blind, placebo-controlled, multicenter phase IV study, FASTLANE by Valeria Rios Rodriguez, Lidia Sánchez-Riera, Hildrun Haibel, Caroline Höppner, Murat Torgutalp, Fabian Proft, Judith Rademacher, Elke Binder, Annette Diehl, Ivana Vranic, Yuxi Zhao, Rajiv Mundayat, Arne Yndestad and Denis Poddubnyy in Therapeutic Advances in Musculoskeletal Disease

## Declarations

**Ethics approval and consent to participate:** The study is performed in accordance with the ethical principles of the Declaration of Helsinki, Good Clinical Practice guidelines, local laws, and regulations and following approval from the IEC/Institutional Review Board (IRB). The study is registered in the EudraCT registry under the number 2023-505050-18-00 and on Clinical Trials.gov with the registration number NCT06112665. Written informed consent must be obtained from each patient before any study-specific procedures are performed. Patients are completely free to refuse to enter the study or to withdraw from it at any time for any reason without incurring any penalty or withholding of treatment on the part of the investigator. The Sponsor has insurance that is in accordance with the legal requirements in countries of conduct. This insurance provides cover for damage to research subjects through injury or death caused by the study. The insurance applies to the damage that occurred during the study or within the timeline specified in the insurance policy and compensation will be provided to the subject in accordance with the policy. The study was fully approved in October 2023 with the first patient’s first visit on November 28, 2023. Approximately 30 sites from Germany, Poland, the Czech Republic, and Bulgaria are participating in the study. The first data results are estimated to be publicly available in the second half of 2025.

**Consent for publication:** Not applicable.

**Competing interests:** V.R.R.: Reports grants from AbbVie, Janssen, Eli Lilly, and UCB; personal fees from AbbVie, Falk, and Takeda; and support for attending meetings and/or travel from Novartis, UCB, and Pfizer outside the presented work. L.S.-R., E.B., A.D., A.Y., R.M., and Y.Z. are Pfizer employees and stockholders of Pfizer Inc. H.H.: Speakers bureau: AbbVie, Janssen, MSD, Novartis, Pfizer, Roche, UCB and Sobi; consultant: Janssen, Novartis, SOBI, and Roche. Research support: SOBI; all outside the presented work. C.H.: Reports support for attending meetings and/or travel from AbbVie outside the presented work. M.T.: Reports support for attending meetings and/or travel from UCB and Janssen outside the presented work. F.P.: Reports grants and personal fees from Novartis, Eli Lilly, and UCB and personal fees from AbbVie, AMGEN, BMS, Celgene, Janssen, Hexal, Medscape, Moonlake, MSD, Pfizer, and Roche outside the presented work. J.R.: Reports support for attending meetings and/or travel from AbbVie, Novartis, and Janssen; and payment for presentations from Janssen outside the presented work. D.P.: Reports grants from Pfizer, AbbVie, Eli Lilly, MSD, Novartis, and Pfizer; personal fees from AbbVie, Biocad, Bristo-Myers Squibb, Eli Lilly, Janssen, Moonlake, Novartis, Pfizer, and UCB; personal speaker fees from AbbVie, Canon, DKSH, Eli Lilly, Janssen, MSD, Medscape, Novartis, Peervoice, Pfizer, and UCB.

**Availability of data and materials:** The study is registered on Clinical Trials.gov with the ID number NCT06112665.
